# Successful Management of Relapsed Severe Immune Thrombocytopenia Using Avatrombopag: A Case Report

**DOI:** 10.1155/crh/2475501

**Published:** 2026-02-05

**Authors:** Sultan Almutairi, Ashraf Warsi, Ahmed Hejazi, Elaf Melibari, Raghad Jar, Amal Almutairi, Asmaa Abughasham, Nuha Firaque

**Affiliations:** ^1^ Department of Adult Haematology, Princess Noorah Oncology Centre, King Abdulaziz Medical City, Ministry of National Guard Health Affairs, Western Region, Jeddah, Saudi Arabia, ngha.med.sa; ^2^ College of Medicine, King Saud Bin Abdulaziz University for Health Sciences, Ministry of National Guard Health Affairs, Western Region, Jeddah, Saudi Arabia, ngha.med.sa; ^3^ King Abdullah International Medical Research Centre, Ministry of National Guard Health Affairs, Western Region, Jeddah, Saudi Arabia, ngha.med.sa

**Keywords:** avatrombopag, bleeding disorders, immune thrombocytopenia, platelet count, thrombopoietin receptor agonists

## Abstract

Immune thrombocytopenia (ITP) is an acquired autoimmune disorder characterized by increased platelet destruction and impaired platelet production. While thrombopoietin receptor agonists (TPO‐RAs), including romiplostim and eltrombopag, have significantly improved ITP management, some patients remain relapsed to multiple lines of therapy, necessitating alternative approaches. Avatrombopag, a second‐generation TPO‐RA, has shown promising efficacy and a favorable safety profile, yet its role in cases unresponsive to prior TPO‐RAs remains underexplored. We report the case of a 37‐year‐old woman with relapsed severe ITP, unresponsive to corticosteroids, IVIG, rituximab, romiplostim, eltrombopag, vincristine, cyclosporine, and splenectomy. Despite multiple treatments, her platelet count remained critically low, with persistent bleeding symptoms. Given the failure of standard therapies, avatrombopag was initiated at 20 mg daily, resulting in a rapid platelet response, increasing from 14 × 10^9^/L to 72 × 10^9^/L within 9 days. The platelet count peaked at 848 × 10^9^/L, necessitating dose adjustments, after which it stabilized within the target range (100–300 × 10^9^/L). The patient tolerated avatrombopag well, with no thromboembolic events or significant adverse effects reported. This case demonstrates the efficacy of avatrombopag in a patient unresponsive to multiple prior therapies, including other TPO‐RAs and splenectomy. Further studies are warranted to determine optimal treatment sequencing and long‐term outcomes for patients in this challenging subgroup.

## 1. Introduction

Primary immune thrombocytopenia (ITP) is an acquired autoimmune disorder characterized by peripheral platelet destruction and impaired platelet production, with an incidence of 2–10 per 100,000 adults [1]. The first‐line treatment aims to restore a hemostatic platelet count using corticosteroids, intravenous immunoglobulin (IVIG), and immunosuppressive agents. Splenectomy is often considered in cases of intolerance or relapse [2]. However, refractory ITP poses a significant risk of life‐threatening bleeding [3], requiring alternative therapeutic strategies [4]. Thrombopoietin receptor agonists (TPO‐RAs), including romiplostim and eltrombopag, have improved treatment outcomes. Still, long‐term efficacy and safety remain challenges, particularly in patients with prior thrombosis or intolerance to therapy [5, 6].

Avatrombopag, a second‐generation TPO‐RA, was approved by the Food and Drug Administration (FDA) in 2019 for chronic ITP with inadequate response to previous treatments. It offers advantages over earlier agents, including no dietary restrictions and a lower risk of hepatotoxicity [7]. We present a case of a 37‐year‐old woman with severe relapsed ITP who failed multiple prior therapies, including steroids, IVIG, rituximab, eltrombopag, romiplostim, vincristine, cyclosporine, and splenectomy, and was successfully managed with avatrombopag. This case highlights the efficacy and safety of avatrombopag as a therapeutic option in relapsed ITP.

## 2. Case Presentation

The ethics approval was waived for this single case report, as they do not constitute “research” involving human subjects under federal regulations, according to the University of Rochester Medical Centre Guideline for Determining Human Subject Research (Page 4 of 8, Final *v*. 21 January 2019). Written informed consent has been obtained from the patient to publish this paper. The manuscript was prepared in accordance with the CARE checklist for case reports (Supporting File 1).

A 37‐year‐old woman presented to Alhada Armed Forces Hospital, Saudi Arabia, with complaints of menorrhagia, fatigue, and extensive bruising. She had a medical history of diabetes mellitus (DM), *Helicobacter pylori*) infection, and hemorrhoids but no family history of a similar hematologic disorder. Her DM was controlled with insulin therapy, and she had previously received clarithromycin and amoxicillin for *H. pylori* eradication. She had also been treated with intravenous (IV) iron for iron deficiency anemia.

Given the persistence of her symptoms and severe thrombocytopenia, a bone marrow biopsy was performed, revealing megakaryocytic hyperplasia and thrombocytopenia consistent with peripheral platelet destruction, leading to a diagnosis of ITP. Additional investigations included an antinuclear antibody (ANA) test, which was positive, raising suspicion for systemic lupus erythematosus (SLE). However, the patient did not meet the full classification criteria for SLE.

The patient was initially treated with high‐dose dexamethasone and IVIG; however, her platelet count remained critically low (< 4 × 10^9^/L). Given her lack of response, treatment was escalated to multiple lines of therapy. She was started on romiplostim at an initial dose of 500 mcg, which was later increased to 750 mcg (10 mcg/kg) due to an inadequate platelet response. Subsequently, she received four doses of rituximab (625 mg each), followed by eltrombopag at 25 mg daily, which was later increased to 50 mg daily when platelet counts remained at 9 × 10^9^/L. A brief trial of combination therapy with rituximab was attempted for 7 days, but it did not achieve a platelet response (platelet count < 10 × 10^9^/L). A subsequent course combining romiplostim and rituximab was administered (10 days in total), which also failed to elicit a meaningful platelet rise (platelet count < 10 × 10^9^/L). Combination therapy was therefore discontinued due to lack of efficacy.

Additional immunosuppressive agents, including azathioprine (100 mg daily), were trialed but later discontinued due to persistent thrombocytopenia (platelet count: 2 × 10^9^/L). Other attempted treatments included dapsone (100 mg daily), cyclosporine (50 mg daily), and vincristine (1 mg), all of which failed to achieve a sustained platelet response.

Due to severe thrombocytopenia and ongoing bleeding risk, an open splenectomy was planned as a definitive intervention. However, the surgery was delayed due to inadequate platelet counts and the high risk of perioperative bleeding, even with platelet transfusions. The patient developed a subarachnoid hemorrhage (SAH) and experienced a tonic‐clonic seizure. She was immediately managed and transferred to the intensive care unit (ICU). At the time of this event, her platelet count was 14 × 10^9^/L, prompting an urgent transfusion of six platelet units, in addition to IVIG (40 g), tranexamic acid (1500 mg IV), and high‐dose methylprednisolone (1000 mg IV once daily for two days). Following stabilization, an open splenectomy was successfully performed without major bleeding complications; the platelet count increased to 200–300 × 10^9^/L immediately after the splenectomy.

Twenty days after splenectomy, the patient presented with rectal bleeding, and laboratory tests revealed persistent thrombocytopenia (platelet count: 15 × 10^9^/L), confirming relapsed ITP despite multiple prior therapies. She was maintained on oral prednisolone (20 mg daily), which was gradually tapered and discontinued.

Due to the failure of conventional treatments, avatrombopag (20 mg daily) was initiated (20 days after splenectomy). Additional investigations, including a nuclear scan to rule out an accessory spleen.

**Figure 1:**
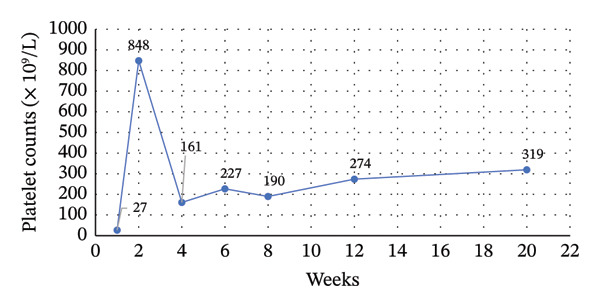
Trend in platelet count Up to 20 weeks after avatrombopag initiation two weeks later, the patient’s platelet count increased to 848 × 10^9^/L, necessitating a temporary discontinuation of avatrombopag for 2 weeks (see Figure 1). A subsequent CBC showed a drop in platelet count to 116 × 10^9^/L, leading to reinitiating avatrombopag at a twice‐weekly dose for 2 weeks. The platelet counts stabilized at 196 × 10^9^/L, allowing further dose reduction to once weekly. Two weeks later, the platelet count reached 277 × 10^9^/L, demonstrating a sustained response with an optimized dosing regimen.

After 9 days of avatrombopag therapy, the patient attended an outpatient follow‐up visit, reporting no new bleeding symptoms or adverse effects. Her complete blood count (CBC) revealed a significant increase in platelet count from 14 × 10^9^/L to 72 × 10^9^/L, prompting the continuation of the same dose with weekly monitoring (Figure 1).

At the latest follow‐up, the patient continues demonstrating stable platelet counts without bleeding episodes and remains clinically stable on a weekly avatrombopag regimen.

## 3. Discussion

The management of ITP is guided by clinical severity, with treatment typically initiated in patients with platelet counts below 20–30 × 10^9^/L to mitigate bleeding risks and enhance quality of life [4]. Relapsed ITP typically refers to patients with persistent thrombocytopenia despite multiple lines of standard treatments or relapse following splenectomy, posing a significant risk of life‐threatening bleeding [8]. The therapeutic objective in relapsed ITP is to maintain a haemostatic platelet count above 20–30 × 10^9^/L while minimising drug‐related toxicity. Corticosteroids are effective in rapidly increasing platelet counts in recently relapsed patients, but adverse effects often limit their long‐term use. Consequently, alternative therapies such as rituximab, a monoclonal anti‐CD20 antibody, and TPO‐RAs have been widely adopted [6]. TPO‐RAs have been linked to higher platelet response rates and reduced need for splenectomy, improving patient quality of life [9].

Combination regimens are often employed for patients who fail to maintain an adequate platelet count with a single therapy [5, 10]. Combinations, such as vinca alkaloids, dapsone, cyclophosphamide, and antimetabolites (e.g., azathioprine, 6‐mercaptopurine, and mycophenolate mofetil [MMF]), have shown variable response rates in relapsed ITP, ranging from 50% to 60% but are often limited by tolerability concerns and relapse after discontinuation. Cyclosporin A has also been explored as a salvage therapy, with a reported response rate of 50%–60%, although 30% of patients discontinued therapy due to adverse effects [5, 11].

Available TPO‐RAs, such as romiplostim, eltrombopag, and avatrombopag, have each shown robust efficacy in adults with chronic ITP, although differences in pivotal trial designs and populations hinder direct comparisons. In a 24 week trial of romiplostim (with parallel cohorts stratified by splenectomy status), durable platelet responses were achieved in ∼60% of non‐splenectomized and ∼38% of splenectomized patients (versus 0%–5% on placebo), with overall platelet response rates around 80%–88% on romiplostim [12]. Similarly, the RAISE trial reported that 79% of patients on eltrombopag achieved a platelet count ≥ 50 × 10^9^/L at least once, significantly higher than the 28% response rate with placebo [13]. The clinical evidence of avatrombopag has been supported by pivotal clinical trials and real‐world evidence. In a phase II trial, 80% of patients in the 20 mg avatrombopag group achieved a platelet response, with 93% responding within the first 7 days [14]. Bhe phase III Core trial confirmed its efficacy, with avatrombopag‐treated patients achieving 12 cumulative weeks oF platelet counts ≥ 50 × 10^9^/L without rescue therapy (*p* < 0.0001) [15]. Notably, the trial populations differed; the romiplostim study enrolled a higher proportion of splenectomised patients (∼50% vs ∼36% in RAISE) and more heavily pretreated cases (63% had ≥ 3 prior ITP therapies vs ∼54% in RAISE). In the RAISE cohort, nearly half the patients were on concurrent ITP medications (stable doses), compared to about one‐third in the romiplostim trials. All three agents were studied in chronic ITP (disease duration ≥ 6 months in most trials), with similar baseline platelet criteria (< 30 × 10^9^/L and ≥ 1 prior therapy in pivotal studies). Despite these population differences, each TPO‐RA produced meaningful platelet count improvements and reduced bleeding rates relative to placebo across their pivotal trials.

Real‐world studies further validated avatrombopag’s effectiveness in diverse patient populations, including those previously treated with other TPO‐RAs or multiple ITP therapies. The REAL‐AVA 1.0 study found that 70% of patients on avatrombopag did not require rescue therapy with corticosteroids, and 93% avoided IVIG use [16]. In comparison, the Spanish ITP Group (GEPTI) study reported a 90.1% platelet response rate among heavily pretreated patients [17]. Additionally, a retrospective study by Al‐Samkari et al. found that 93% of patients who transitioned from romiplostim or eltrombopag to avatrombopag achieved a platelet response, with 86% achieving a complete response [18].

In our case, the failure of both TPO‐RAs (romiplostim and eltrombopag) highlights the heterogeneity in individual treatment responses and underscores the clinical challenge of managing relapsed ITP [19]. In our case, avatrombopag led to a rapid platelet response in the present case within 9 days, increasing counts from 14 × 10^9^/L to 72 × 10^9^/L. Moreover, the patient’s platelet counts peaked at 848 × 10^9^/L, highlighting the dose‐dependent response observed and reinforcing the importance of careful monitoring to prevent excessive thrombocytosis. Additionally, in alignment with real‐world data showing steroid‐sparing benefits, our case was able to discontinue corticosteroids following avatrombopag initiation, reducing the risk of long‐term steroid‐related complications.

In contextualizing our patient’s response to avatrombopag, it is important to compare with the published clinical‐trial data and available cohort analyses for postsplenectomy patients with chronic ITP. In a large analysis of romiplostim trials, for example, 82% of non‐splenectomized patients achieved a platelet response (≥ 50 × 10^9^/L) versus only ∼68% of splenectomized patients, and sustained response rates were similarly higher in those without splenectomy (80% vs. 68%) [20]. Although subgroup data specifically for postsplenectomy patients treated with avatrombopag are not available, a meta‐analysis TPO‐RAs reports that splenectomized patients have a somewhat lower durable response than nonsplenectomized when analyzed collectively for TPO‐RA therapy [21]. One possible explanation is that patients requiring splenectomy represent a more refractory subset of ITP, with a longer disease duration, lower baseline platelet counts, and having received more prior therapies [20]. In addition, splenectomy itself may introduce changes in disease pathogenesis that make relapsed ITP harder to treat. The spleen is a primary site of platelet destruction and autoantibody production in ITP [22]. Splenectomy can alter the immune mechanisms of ITP, and persistent ITP may suggest that the immune system has shifted to other sites for platelet destruction and antibody generation. Notably, refractory ITP patients are often enriched for pathological autoantibodies such as anti‐GPIb/IX. These antibodies can mediate platelet clearance through Fc‐independent mechanisms [23]. There is also evidence that cell‐mediated immunity (e.g., cytotoxic T‐lymphocytes targeting platelets or megakaryocytes) contributes to refractory ITP, a pathogenic mechanism that would not be alleviated by splenectomy [23].

In the present case, the patient demonstrated persistent thrombocytopenia despite multiple combinations of standard treatments. Initial therapies, including corticosteroids and IVIG, failed to elicit a sustained response, necessitating escalation to rituximab, eltrombopag, romiplostim, vincristine, cyclosporine, and eventually splenectomy. Despite these interventions, postsplenectomy relapse occurred, confirming the patient’s refractory status.

Avatrombopag is generally well‐tolerated in chronic ITP, with most adverse events (AEs) being mild and transient. Thromboembolic events occurred in 7% of patients in clinical trials, primarily in those with pre‐existing risk factors [7]. Real‐world data from the Spanish ITP Group (GEPTI) study found a 4.5% thrombosis rate, including pulmonary embolism, ischemic stroke, and myocardial infarction [17]. A case of catastrophic antiphospholipid syndrome following avatrombopag use highlights the need for caution in high‐risk patients [24]. Unlike eltrombopag, avatrombopag does not cause significant hepatotoxicity and does not require routine liver monitoring. Liver enzyme elevations occurred in 5.5% of patients but resolved without discontinuation. Gastrointestinal toxicity is minimal, with no reported cases of gastric atrophy or bone marrow suppression [7]. In this case, no thromboembolic events were observed despite a platelet surge to 848 × 10^9^/L. The patient tolerated avatrombopag without significant AEs, supporting its favorable safety profile in relapsed ITP.

## 4. Conclusions

This case highlights the efficacy of avatrombopag in relapsed ITP, particularly in a patient unresponsive to multiple prior therapies, including romiplostim, eltrombopag, and rituximab, despite undergoing splenectomy. Avatrombopag led to a rapid and sustained platelet increase following the failure of other TPO‐RAs, reinforcing its potential role in treatment sequencing for relapsed ITP. Given its favorable safety profile and robust platelet response, avatrombopag represents a valuable alternative for patients who have exhausted other therapeutic options, warranting further studies to define optimal treatment strategies in this setting.

## Funding

Sobi provided funding for the medical writing activities of this manuscript through a medical grant. The sponsor did not influence the content or direction of the manuscript but was allowed to review it as a courtesy.

## Disclosure

All authors have read and approved the final version of the manuscript. Dr Sultan Almutairi had full access to all of the data in this study and takes complete responsibility for the integrity of the data and the accuracy of the data analysis.

## Conflicts of Interest

The authors declare no conflicts of interest.

## Supporting Information

Supporting file 1: CARE checklist for case reports.

## Supporting information


**Supporting Information** Additional supporting information can be found online in the Supporting Information section.

## Data Availability

The data that support the findings of this study are available on request from the corresponding author. The data are not publicly available due to privacy or ethical restrictions.
